# Climate change and Australian general practice vocational education: a cross-sectional study

**DOI:** 10.1093/fampra/cmac053

**Published:** 2022-05-25

**Authors:** Kathleen Wild, Amanda Tapley, Alison Fielding, Elizabeth Holliday, Jean Ball, Graeme Horton, Grant Blashki, Andrew Davey, Mieke van Driel, Alexandria Turner, Kristen FitzGerald, Neil Spike, Parker Magin

**Affiliations:** School of Medicine and Public Health, University of Newcastle, University Drive, Callaghan, Newcastle, NSW 2308, Australia; School of Medicine and Public Health, University of Newcastle, University Drive, Callaghan, Newcastle, NSW 2308, Australia; NSW & ACT Research and Evaluation Unit, GP Synergy Regional Training Organisation (RTO), 20 McIntosh Drive, Mayfield West, NSW 2304, Australia; School of Medicine and Public Health, University of Newcastle, University Drive, Callaghan, Newcastle, NSW 2308, Australia; NSW & ACT Research and Evaluation Unit, GP Synergy Regional Training Organisation (RTO), 20 McIntosh Drive, Mayfield West, NSW 2304, Australia; School of Medicine and Public Health, University of Newcastle, University Drive, Callaghan, Newcastle, NSW 2308, Australia; Clinical Research Design and Statistical Support Unit (CReDITSS), John Hunter Hospital, Hunter Medical Research Institute (HMRI), New Lambton Heights, NSW 2305, Australia; School of Medicine and Public Health, University of Newcastle, University Drive, Callaghan, Newcastle, NSW 2308, Australia; Nossal Institute and the Melbourne Sustainable Society Institute, The University of Melbourne, Parkville, VIC 3010, Australia; School of Medicine and Public Health, University of Newcastle, University Drive, Callaghan, Newcastle, NSW 2308, Australia; NSW & ACT Research and Evaluation Unit, GP Synergy Regional Training Organisation (RTO), 20 McIntosh Drive, Mayfield West, NSW 2304, Australia; General Practice Clinical Unit, Faculty of Medicine, Royal Brisbane & Women’s Hospital, University of Queensland, Level 8, Health Sciences Building, Brisbane, QLD 4029, Australia; School of Medicine and Public Health, University of Newcastle, University Drive, Callaghan, Newcastle, NSW 2308, Australia; NSW & ACT Research and Evaluation Unit, GP Synergy Regional Training Organisation (RTO), 20 McIntosh Drive, Mayfield West, NSW 2304, Australia; General Practice Training Tasmania Regional Training Organisation, Level 3, RACT House, 179 Murray Street, Hobart, TAS 7000, Australia; Tasmanian School of Medicine, University of Tasmania, Level 1, Medical Science 1, 17 Liverpool Street, Hobart, TAS 7000, Australia; Eastern Victoria General Practice Training Regional Training Organisation, 15 Cato Street, Hawthorn, VIC 3122, Australia; Department of General Practice and Primary Health Care, University of Melbourne, 200 Berkeley Street Carlton, VIC 3053, Australia; Faculty of Medicine, Nursing and Health Sciences, School of Rural Health, Monash University, Northways Road, Churchill, VIC 3842, Australia; School of Medicine and Public Health, University of Newcastle, University Drive, Callaghan, Newcastle, NSW 2308, Australia; NSW & ACT Research and Evaluation Unit, GP Synergy Regional Training Organisation (RTO), 20 McIntosh Drive, Mayfield West, NSW 2304, Australia

**Keywords:** climate change, eco-health, eco-medical literacy, general practice education, sustainable healthcare

## Abstract

**Background:**

Climate change is a rapidly progressing threat to global health and well-being. For general practitioners (GPs) currently in training, the effects of climate change on public health will shape their future professional practice We aimed to establish the prevalence and associations of Australian GP registrars’ (trainees’) perceptions of climate change as it relates to public health, education, and workplaces.

**Methods:**

A cross-sectional questionnaire-based study of GP registrars of three Australian training organizations. The questionnaire assessed attitudes regarding adverse health effects of climate change (over the next 10–20 years), and agreement with statements on (i) integrating health impacts of climate change into GP vocational training, and (ii) GPs’ role in making general practices environmentally sustainable.

**Results:**

Of 879 registrars who participated (response rate 91%), 50.4% (95% CI 46.8%, 54.0%) perceived a large or very large future health effect of climate change on their patients, and 61.8% (95% CI 58.6%, 65.0%) agreed that climate health impacts should be integrated within their education programme. 77.8% (95% CI 74.9%, 80.4%) agreed that GPs should have a leadership role in their practices’ environmental sustainability. Multivariable associations of these attitudes included female gender, training region, and (for the latter two outcomes) perceptions of future impact of climate change on patient health.

**Conclusions:**

GP registrars are motivated to receive climate health education and engage in environmentally sustainable practice. This may primarily reflect concern for future practice and patient care.

**Key messages**

- Climate change has significant implications for medical practice.
- Climate and environmental health impacts can be integrated into medical education.
- Australian general practice trainees recognize health impacts of climate change.
- Australian general practice trainees recognize need for climate health education.
- General practice educators should develop climate health learning material.

## Introduction

Climate change is a rapidly progressing threat to health and well-being.^1^ Health effects to be observed in general practice include increased incidence of heat stress,^2,3^ allergic disease (due to increased levels of airborne pollen and mould allergens),^4,5^ and mental health distress secondary to climate change-related factors, including extreme weather events and community displacement.^6^ Climate change-related extreme temperature events are associated with cardiovascular and respiratory morbidity and mortality as well as with overall mortality.^7^

For Australian general practice (GP) registrars (trainees) at the start of their careers, the dynamics of climate change on public health will shape their future professional practice. Not only will the clinical presentations and outcomes for patients change, but also the form and function of medical workplaces. Recent events in Australia have shown adverse health events attributable to climate change, including the significant wave of air pollution-related disease from a catastrophic 2019–2020 bushfire season^8^ and the unprecedented 2022 East Coast floods.^9^ This has highlighted the importance of action to ensure health professionals are prepared to respond to the public health threats of climate change.

Over the last decade, integrating climate change with medical curricula has been a challenge for educators as the breadth of topics required to teach future professionals has expanded.^10^ The Royal Australian College of General Practitioners (RACGP) and Australian College of Rural and Remote Medicine (ACRRM) have formally included the need to understand the health impacts of environmental change in College curricula.^11,12^ It has been less clear, however, how educators could integrate climate education into registrar teaching within the context of GP vocational training. The imperative falls to educators to create novel methods of integrating climate change literacy into the crowded postgraduate medical curriculum. Climate change education and environmental health literacy have gained momentum in recent years, and undergraduate climate curriculum frameworks have been developed.^13,14^

Compared to the work in medical school curricula, there has been less development of climate education frameworks for postgraduate learners. Responding to the perceived learning needs of adult learners in specialty training programmes can be used to build a curriculum.^15^ In this study, we aimed to establish the perceptions of GP registrars in Australia towards climate change as a public health concern (over the next 10–20 years), learning need, and workplace issues.

## Methods

### Study design

This was a cross-sectional questionnaire-based study nested within the Registrar Clinical Encounters in Training (ReCEnT) project.

### ReCEnT

ReCEnT is an ongoing cohort study of the in-consultation clinical and educational experiences of GP registrars,^16^ conducted in three Australian Regional Training Organisations training 43% of Australia’s registrars.^17^ ReCEnT is an integral part of registrars’ training,^18,19^ and registrars may consent to their data also being used for research purposes.

During each of three 6-month GP training terms participating registrars complete a questionnaire establishing characteristics of the registrars and their current training practice. This questionnaire was used in the current cross-sectional analysis. Consultation data (also collected in ReCEnT) were not used.

### Outcome factors

Three items related to climate change were included in the early-2019 ReCEnT questionnaire. The first item assessed the perceived significance of climate change as a health issue: “The adverse health effects of climate change on my patients in the next 10–20 years will be:”. Options were a five-point scale from “Nil” to “Very Large” with an additional option to select “Don’t know”. Participants were also asked to indicate agreement with the following statements on a five-point Likert scale: “Teaching about the health impacts of climate change should be integrated into GP vocational training education programs” and “GPs should have a leadership role in encouraging their general practices to be as environmentally sustainable as possible”.

The items were developed from selected survey questions used by Sarfaty et al. in multiple surveys of various US and global physician populations regarding their attitudes to climate change,^20–22^ adapted to our study population.

For analysis, responses to the three items were dichotomized. Responses to the first item regarding perceived future health impact of climate change were dichotomized with “Nil”, “Small”, and “Moderate” compared to “Large” and “Very Large”. Dichotomization was based on our assessment of the literature that, in 10–20 years, climate effects on health will be at least “large”.^3^ For analyses of this outcome, responses of “don’t know” were excluded.

Responses to the questions “Teaching about the health impacts of climate change should be integrated into GP vocational training education programs” and “GPs should have a leadership role in encouraging their general practices to be as environmentally sustainable as possible” were dichotomized with “Strongly Disagree”, “Disagree”, and “Neutral” compared to “Agree” and “Strongly Agree”.

### Independent variables

Independent registrar level and practice level variables were used in the analyses. Registrar level variables were: age; gender; part- or full-time employment; training term; country of primary medical qualification; training region; previous health or non-health qualification; and worked at current practice before. Practice variables were: practice size; whether the practice bulk-bills (no fee payable by the patient) for all patients; level of rurality (defined by the Australian Standard Geographical Classification-Remoteness Area [ASGC-RA]),^23^ and socioeconomic status of the practice location (defined by Socio-Economic Indexes for Areas, Index of Relative Socioeconomic Disadvantage).^24^

For analyses of the second and third outcomes, “perceived future health impact of climate change” was also an independent variable.

### Statistical analyses

The proportions of registrars who perceived that future health impact of climate change was large/very large, who agreed/strongly agreed that climate change should be integrated into GP vocational training education programmes, and who agreed/strongly agreed with GPs having a leadership role in environmentally sustainable general practices were calculated, together with 95% CI.

Descriptive statistics included frequencies for categorical variables and means with SD for continuous variables.

Univariate logistic regressions were conducted on each covariate with each outcome. Covariates with a univariate *P*-value <0.20 were considered for inclusion in the multiple regression model.

Once multivariable logistic regressions with all significant covariates were fitted, model reduction was assessed on each model. Covariates that were no longer significant (at *P *< 0.2) in the multivariable model were tested for removal from the model. If the covariate’s removal did not substantively change the resulting model, the covariate was removed from the final model. Substantive change was defined as any covariate changing in the effect size (odds ratio) of greater than 10%.

Diagnostic tests were conducted to assess goodness of fit, using the Hosmer–Lemeshow test for logistic models. Associations were considered significant at the 0.05 level.

## Results

A total of 879 registrars participated, of 966 who received the survey (response rate 91.0%).

Characteristics of participating registrars are presented in Table 1.

**Table 1.** Characteristics of registrar respondents.

Variable	Class	*n* (%)
Registrar gender	Male	413 (42.1)
	Female	568 (57.9)
Registrar age	Mean (SD)	33.2 ± 6.5
Registrar full- or part-time employment	Full-time	672 (73.8)
	Part-time	239 (26.2)
Term of training	Term 1	458 (46.5)
	Term 2	126 (12.8)
	Term 3	401 (40.7)
Training region	Region 1	140 (14.2)
	Region 2	48 (4.9)
	Region 3	202 (20.5)
	Region 4	323 (32.8)
	Region 5	272 (27.6)
Primary qualification as doctor in Australia	No	224 (22.8)
	Yes	760 (77.2)
Health qualification prior to medical qualification	No	844 (86.8)
	Yes	128 (13.2)
Non-health qualification prior to medical qualification	No	656 (67.6)
	Yes	314 (32.4)
Worked at practice previously	No	829 (85.6)
	Yes	139 (14.4)
Practice always bulk-bills^a^	No	622 (63.3)
	Yes	361 (36.7)
Practice size	Small	389 (42.5)
	Large	526 (57.5)
Rurality classification	Major city	582 (59.7)
	Inner regional	305 (31.3)
	Outer regional/remote/very remote	88 (9.0)
SEIFA-IRSD^b^	mean (SD)	5.4 ± 2.7

^a^Patient fee entirely subsidized by government.

^b^Socio-Economic Indexes for Areas, Index of Relative Socioeconomic Disadvantage.

**Fig. 1.**
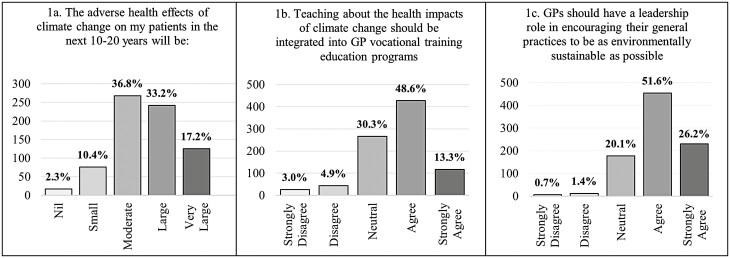
Responses to study questions.

Registrar responses to the three questionnaire items are presented in Fig. 1.

For the question regarding perceived adverse health effects of climate change, there were 728 responses (after the exclusion of 150 responses of “Don’t Know”). Of these, 50.4% perceived a “Large” or “Very Large” effect (95% CI 46.8%, 54.0%).

To the statement that teaching health impacts of climate change should be integrated with GP vocational training education, 61.8% (95% CI 58.6%, 65.0%) agreed or strongly agreed.

For the statement regarding GP leadership on sustainability within their practices, 77.8% (95% CI 74.9%, 80.4%) agreed or strongly agreed.

Characteristics associated with perceived large or very large climate effects on future patient health are presented in Supplementary Table 1. Results of univariate and multivariable logistic regressions are presented in Table 2. On multivariable analysis, perception of large/very large effects was significantly associated only with training region (OR 1.89; 95% CI 1.13, 3.18; *P *= 0.016 and 1.75; 95% CI 1.05, 2.89; *P *= 0.03) for two regions compared to the reference region). Goodness-of-fit tests showed the model was a good fit (χ^2^ = 2.76, *P *= 0.84).

**Table 2.** Model: Associations with perception of adverse impacts of climate change on health.

Factor group	Variable	Class	Univariate		Adjusted	
			OR (95% CI)	*P*	OR (95% CI)	*P*
Registrar factors	Gender	Female	1.22 (0.91, 1.64)	0.19	1.23 (0.91, 1.66)	0.18
	Training region (Referent: Region 1)	Region 2	1.77 (0.83, 3.78)	0.15	1.83 (0.85, 3.91)	0.12
		Region 3	1.86 (1.11, 3.12)	0.018	1.89 (1.13, 3.18)	0.016
		Region 4	1.01 (0.62, 1.66)	0.96	1.03 (0.63, 1.70)	0.90
		Region 5	1.72 (1.04, 2.85)	0.034	1.75 (1.05, 2.89)	0.030

Characteristics associated with strong/very strong belief that health impacts of climate change should be integrated into GP vocational education are presented in Supplementary Table 2. Results of univariate and multivariable logistic regressions are presented in Table 3. On multivariable analysis, there were significant associations with female gender (OR 1.84; 95% CI 1.31, 2.59; *P *= 0.005)), perceived large or very large climate effects on future patient health (OR 4.19; 95% CI 2.95, 5.96; *P* = <0.001), and training region (OR 0.49; 95% CI 0.26, 0.91; *P *= 0.024 and 0.53; 95% CI 0.29, 0.96; *P *= 0.04) for two regions compared to the reference region). Goodness-of-fit tests showed the model was a good fit (χ^2^ = 5.78, *P *= 0.67).

**Table 3.** Model: Associations with agreement that health impacts of climate change should be integrated with GP vocational training education.

Factor group	Variable	Class	Univariate		Adjusted	
			OR (95% CI)	*P*	OR (95% CI)	*P*
Registrar factors	Gender	Female	1.61 (1.22, 2.11)	<0.001	1.84 (1.31, 2.59)	<0.001
	Age		1.02 (1.00, 1.04)	0.076	1.02 (0.99, 1.04)	0.25
	Training region (Referent: Region 1)	Region 2	1.90 (0.85, 4.22)	0.12	1.67 (0.59, 4.73)	0.34
		Region 3	0.82 (0.51, 1.32)	0.41	0.49 (0.26, 0.91)	0.024
		Region 4	0.65 (0.41, 1.02)	0.060	0.53 (0.29, 0.96)	0.035
		Region 5	0.91 (0.57, 1.45)	0.69	0.65 (0.35, 1.19)	0.16
	Response regarding adverse effects of climate change on health	Larger effect	4.09 (2.92, 5.74)	<0.001	4.19 (2.95, 5.96)	<0.001

Characteristics associated with strong/very strong belief that GPs should lead environmental sustainability within practices are presented in Supplementary Table 3. Results of univariate and multivariable logistic regressions are presented in Table 4. On multivariable analysis, there were significant associations with training in a larger practice (OR for smaller practice 0.61; 95% CI 0.40, 0.93; *P *= 0.02) and perceived large/very large climate effects on future patient health (OR 4.56; 95% CI 2.87, 7.24, *P* = <0.001). There was some evidence for an association with female gender (OR 1.49; 95% CI 0.99, 2.25; *P *= 0.06). Again, there was variability between geographic regions, but comparisons with the referent region were not significant at the *P *< 0.05 level. Goodness-of-fit tests showed the model was a good fit (χ^2^ = 8.57, *P *= 0.38).

**Table 4.** Model: Associations with agreement that GPs hold leadership role in practice sustainability.

Factor group	Variable	Class	Univariate		Adjusted	
			OR (95% CI)	*P*	OR (95% CI)	*P*
Registrar factors	Gender	Female	1.66 (1.20, 2.28)	0.002	1.49 (0.99, 2.25)	0.055
	Qualified as doctor in Australia	Yes	0.53 (0.34, 0.82)	0.004	0.76 (0.43, 1.35)	0.36
	Training region	Region 3	1.62 (0.61, 4.28)	0.33	1.50 (0.42, 5.34)	0.53
		Region 4	1.06 (0.59, 1.91)	0.85	0.81 (0.37, 1.79)	0.60
		Region 6	0.59 (0.35, 1.01)	0.052	0.50 (0.22, 1.11)	0.089
		Region 7	0.87 (0.50, 1.52)	0.63	1.05 (0.49, 2.25)	0.90
	Response regarding adverse effects of climate change on health	Larger effect	4.70 (3.00, 7.37)	<0.001	4.56 (2.87, 7.24)	<0.001
Practice factors	Practice size	Small	0.77 (0.56, 1.06)	0.11	0.61 (0.40, 0.93)	0.021
	Rurality	Inner regional	1.12 (0.79, 1.60)	0.51	0.62 (0.32, 1.19)	0.15
		Outer regional/remote/very remote	1.95 (1.00, 3.81)	0.049	1.13 (0.42, 3.03)	0.81

## Discussion

This is the first study of GP registrar (or of GP trainee in any setting) attitudes regarding climate change education. Half of the respondents felt climate change would have a large or very large effect on future patients’ health. We found high registrar engagement with the issue, with strong agreement that climate change should be integrated into the curriculum and that GPs should lead practice sustainability. There was some geographic variation, and some associations of registrar characteristics (female gender for education) and practice characteristics (larger practice size regarding leadership) with these attitudes. But the strongest factor in agreement regarding the curriculum or practice leadership was the opinion that climate change would have adverse patient health impacts.

### Comparison to previous literature

While attitudes of GP registrar/trainee populations towards climate health and education have not been previously assessed, studies have been conducted in other physician and student populations.

Horton explored the attitudes of Australian medical students to climate change education in a mixed-methods study.^10^ A survey of Australian medical students identified that 85.2% considered climate change a health threat, and that 82.6% agreed on the importance of medical education on the health impacts of climate change. In a subsequent qualitative study, Horton also interviewed educators to explore the implementation of undergraduate climate curriculums. While many acknowledged the impacts of climate change, there was scepticism that these effects would be felt locally and some reluctance to add another “issue” to the curriculum. However, the relevance to a Public Health curriculum was recognized by many participating educators.

Sarfaty et al. conducted multiple studies investigating the attitudes of international physician association members.^22^ Across different physician groups, agreement regarding inclusion of climate change into continuing medical education (CME) curricula ranged from 67% to 89%.^21,22^ The registrars in our study had slightly less agreement regarding integration of climate change in registrar education compared to the physician opinion regarding CME. Context for these findings is that GP registrars are training within a relatively short vocational programme with major summative examinations. It is possible they prioritize learning examinable content and perceive climate effects to be less likely to be examined. This is of relevance as engaging the adult learner must consider their motivation.^25^

In the Australian GP context, practitioner attitudes have been explored in a study investigating the attitudes of rural Australian GPs towards disaster preparedness.^26^ This was a small study of 68 practitioners in which there was agreement that climate change was likely to impact a range of future health outcomes.

### Strengths and limitations

This is, to the best of our knowledge, the first study looking at the attitudes of GP trainees towards environmental impacts on patient health, trainee education, and the workplace. The response rate of 91.0% (very high for surveys of general practitioners^27^) is a strength, as are the characteristics of the participating registrars (which make findings generalizable to the Australian GP training context, with probable generalizability to other countries with similar health systems and GP training programmes).

A limitation of this study is that complexity is difficult to capture in three survey items, and further research is needed to give more depth to how GP trainees, and other stakeholders in GP education, understand the need for eco-medical literacy. Different methodological approaches, such as the mixed-methods study of undergraduate medical students employed by Horton,^10^ may better capture the nuance of to approach this topic in medical education.

### Implications

In Australia, the colleges governing GP education include environmental impacts on health in their training curricula. The RACGP refers to “climate change and its consequences” as a key risk factor affecting disease prevalence,^11^ and the ACRRM Primary Curriculum 2013 refers to “the social, environmental, economic and occupational determinants of health”.^28^

While all curriculum expansion occurs in a crowded space occupied by pre-existing stakeholders, the complex manifestations of climate change on human health lead to the possibility of integrating climate education with topics across multiple domains to build an overall understanding of “eco-medical literacy”.^29^ This could include education on specific clinical entities (i.e. asthma, infectious disease, or mental health), population health and epidemiology, and Indigenous health.

The challenge is translation into meaningful education for GP registrars. Most work regarding the climate curriculum has centred on undergraduate medical education^30,31^; including an Australasian working group established in 2019 by medical school deans to develop a curriculum framework about climate change and its impacts on health systems.^32^ In much published work on improving understanding in the medical profession of environmental impacts on health, the contention has been that integrating climate change with existing curricula topics is the ideal way to deliver this education (compared to standalone education modules). Suggestions for how this integration could be implemented have been put forward by Maxwell and Blashki,^33^ who see a positive opportunity in climate education to improve the depth of medical education across multiple domains. While it is critical to build the capacity of future doctors to respond to the changing environment, we must ensure that this process does not stop as graduates enter the workforce. Resources must be developed to meet the needs of postgraduate learners across all medical specialities regarding climate change. While previous investigations have identified caution in groups of medical educators and policymakers regarding the relevance of this topic to medical education,^10^ our findings suggest that GP registrars see climate change as highly relevant to their practice.

The models of climate change and sustainability education of medical schools could be a foundation to develop teaching that meets the needs of postgraduate trainees and their patients, recognizing the need for adaptation given the differing structures of the training environments.

We found significant associations with region for two of our outcomes, despite there being no significant association with level of rurality. This regional heterogeneity in attitudes may have educational implications. Climate change education in this trainee population could be centred on the impacts of climate change on local communities, and the required response of health practitioners and institutions.

Recent events in Australian communities affected by natural disasters such as fire and flood have demonstrated that GPs have a role to play in disaster recovery efforts. Understanding of climate change and its effects can help practitioners anticipate future regional public health trends and needs. Responsiveness to community needs is a hallmark of primary care and could be actively demonstrated by addressing local climate impacts. This can also extend to GPs playing a role in policy development for a more coherent long-term local health system response to climate change.

Our findings that “belief that health impacts of climate change should be integrated into GP vocational education” and “GPs should have a role in encouraging environmental sustainability within their practices” were both strongly associated with the perception of large or very large climate effects on future patients’ health are notable. This may suggest that early-career GPs’ receptivity to education and preparedness to accept responsibility related to environmental sustainability is strongly driven by specific concerns for future practice and future patients. It is arguable that this may be a more sustainable motivation for action than a more general concern for climate change.

Awareness of environmental factors could also prepare practitioners to operate health services resilient to the challenges of climate change. Skill-building in sustainable health services was raised as a learning objective for medical students in an international collaboration,^31^ specifically regarding healthcare system delivery, quality improvement processes, and resource management. These skills are more relevant to registrars in the workforce than medical students. Embedding these skills within the registrar training programme could improve the professional capacity of the workforce to adapt to climate conditions and mitigate the effect of healthcare on greenhouse gas emissions.

Our findings suggest that the registrar workforce places a high value on the environmental sustainability of their workplace, and that there is little risk of alienating trainees by developing higher sustainability standards in Australian general practice. In other employment settings, sustainability has been connected to employee satisfaction and well-being.^34,35^ Employees who value environmental sustainability are more content in workplaces that respond in kind. For employers, augmenting workplace sustainability may improve employee satisfaction and retention. Further research and engagement in community and hospital settings may help to clarify the benefits for physician well-being arising from workplace sustainability.

## Conclusions

We found high motivation in GP registrars for climate change and sustainability education and practice. Further research could ascertain whether these attitudes are consistent across other GP populations, and gain a more detailed understanding of practitioner attitudes towards the intersection of general practice, climate change, and environmental sustainability. This understanding could deliver curriculum development for adult learning in the registrar population as well as appropriate CME for GPs. Support for practices to reduce their environmental impact may also lead to improved professional and management outcomes.

These interventions could aid general practitioners to respond and adapt to the public health threat of climate change, and lead to a reduction in the impact that Australian general practice has on climate pollution.

## Supplementary Material

cmac053_suppl_Supplementary_Table_S1

cmac053_suppl_Supplementary_Table_S2

cmac053_suppl_Supplementary_Table_S3

cmac053_suppl_Supplementary_Checklist

## Data Availability

The data underlying this article cannot be shared publicly due to advice of the relevant Human Research Ethics Committee.
