# Supplementary material for: Climate change and Australian general practice vocational education: a cross-sectional study
Source: Fam Pract. 2022 May 25;40(3):435–41. doi: 10.1093/fampra/cmac053 (PMC10231347; doi:10.1093/fampra/cmac053)
Supplement: cmac053_suppl_Supplementary_Table_S1 [file cmac053_suppl_supplementary_table_s1.docx]

### Supplementary Table 1. Associations with perception of adverse impacts of climate change on health

| **Factor group** | **Variable** | **Class** | **Nil / Small/ Moderate** | **Large / Very Large** | **p** |
| --- | --- | --- | --- | --- | --- |
| Registrar factors | Gender | Male | 158 (44%) | 143 (39%) | 0.18 |
|  |  | Female | 203 (56%) | 224 (61%) |  |
|  | Full or part-time employment | Part-time | 97 (27%) | 95 (26%) | 0.71 |
|  |  | Full-time | 258 (73%) | 269 (74%) |  |
|  | Term of training | Term 1 | 176 (49%) | 172 (47%) | 0.85 |
|  |  | Term 2 | 44 (12%) | 49 (13%) |  |
|  |  | Term 3 | 141 (39%) | 146 (40%) |  |
|  | Primary qualification as doctor in Australia | No | 88 (24%) | 84 (23%) | 0.64 |
|  |  | Yes | 273 (76%) | 283 (77%) |  |
|  | Health qualification before medical qualification | No | 312 (87%) | 324 (89%) | 0.57 |
|  |  | Yes | 46 (13%) | 42 (11%) |  |
|  | Non-health qualification before medical qualification | No | 244 (68%) | 245 (67%) | 0.69 |
|  |  | Yes | 114 (32%) | 122 (33%) |  |
|  | Training region | Region 1 | 52 (14%) | 38 (10%) | 0.006 |
|  |  | Region 3 | 17 (5%) | 22 (6%) |  |
|  |  | Region 4 | 72 (20%) | 98 (27%) |  |
|  |  | Region 6 | 131 (36%) | 97 (26%) |  |
|  |  | Region 7 | 89 (25%) | 112 (31%) |  |
|  | Worked at practice previously | No | 297 (84%) | 307 (85%) | 0.67 |
|  |  | Yes | 58 (16%) | 55 (15%) |  |
|  | Age | mean (SD) | 33 (7) | 33 (6) | 0.72 |
| Practice factors | Always bulk-bills | No | 215 (60%) | 225 (61%) | 0.60 |
|  |  | Yes | 146 (40%) | 141 (39%) |  |
|  | Practice size | Small | 163 (46%) | 150 (41%) | 0.25 |
|  |  | Large | 195 (54%) | 213 (59%) |  |
|  | Rurality | Major city | 221 (62%) | 204 (56%) | 0.36 |
|  |  | Inner regional | 105 (29%) | 121 (33%) |  |
|  |  | Outer regional/remote/very remote | 33 (9%) | 37 (10%) |  |
|  | SEIFA-IRSD | mean (SD) | 5 (3) | 5 (3) | 0.30 |
