# Supplementary material for: Climate change and Australian general practice vocational education: a cross-sectional study
Source: Fam Pract. 2022 May 25;40(3):435–41. doi: 10.1093/fampra/cmac053 (PMC10231347; doi:10.1093/fampra/cmac053)
Supplement: cmac053_suppl_Supplementary_Table_S2 [file cmac053_suppl_supplementary_table_s2.docx]

### Supplementary Table 2. Associations with agreement that health impacts of climate change should be integrated with GP vocational training education

| **Factor group** | **Variable** | **Class** | **Disagree** | **Agree** | **p** |
| --- | --- | --- | --- | --- | --- |
| Registrar factors | Gender | Male | 165 (49%) | 205 (38%) | <0.001 |
|  |  | Female | 170 (51%) | 339 (62%) |  |
|  | Full or part time employment | Part time | 85 (26%) | 147 (27%) | 0.61 |
|  |  | Full-time | 245 (74%) | 391 (73%) |  |
|  | Term of training | Term 1 | 159 (47%) | 262 (48%) | 0.27 |
|  |  | Term 2 | 33 (10%) | 71 (13%) |  |
|  |  | Term 3 | 143 (43%) | 211 (39%) |  |
|  | Primary qualification as doctor in Australia | No | 53 (16%) | 140 (26%) | <0.001 |
|  |  | Yes | 282 (84%) | 404 (74%) |  |
|  | Health qualification before medical qualification | No | 288 (86%) | 479 (89%) | 0.37 |
|  |  | Yes | 45 (14%) | 62 (11%) |  |
|  | Non-health qualification before medical qualification | No | 221 (66%) | 369 (68%) | 0.57 |
|  |  | Yes | 112 (34%) | 172 (32%) |  |
|  | Training Region | Region 1 | 39 (12%) | 76 (14%) | 0.027 |
|  |  | Region 3 | 10 (3%) | 37 (7%) |  |
|  |  | Region 4 | 76 (23%) | 121 (22%) |  |
|  |  | Region 6 | 122 (36%) | 154 (28%) |  |
|  |  | Region 7 | 88 (26%) | 156 (29%) |  |
|  | Worked at practice previously | No | 285 (87%) | 446 (83%) | 0.20 |
|  |  | Yes | 44 (13%) | 89 (17%) |  |
|  | Response to question regarding Adverse effects of climate change on health | Smaller effect | 169 (72%) | 192 (39%) | <0.001 |
|  |  | Larger effect | 65 (28%) | 302 (61%) |  |
|  | Age | mean (SD) | 33 (6) | 33 (7) | 0.076 |
| Practice factors | Always bulk-bills | No | 206 (61%) | 331 (61%) | 0.87 |
|  |  | Yes | 129 (39%) | 212 (39%) |  |
|  | Practice size | Small | 150 (45%) | 223 (41%) | 0.25 |
|  |  | Large | 181 (55%) | 316 (59%) |  |
|  | Rurality | Major city | 221 (66%) | 297 (55%) | 0.003 |
|  |  | Inner regional | 93 (28%) | 179 (33%) |  |
|  |  | Outer regional/remote/very remote | 20 (6%) | 60 (11%) |  |
|  | SEIFA-IRSD | mean (SD) | 6 (3) | 5 (3) | 0.19 |
