# Supplementary material for: Climate change and Australian general practice vocational education: a cross-sectional study
Source: Fam Pract. 2022 May 25;40(3):435–41. doi: 10.1093/fampra/cmac053 (PMC10231347; doi:10.1093/fampra/cmac053)
Supplement: cmac053_suppl_Supplementary_Table_S3 [file cmac053_suppl_supplementary_table_s3.docx]

### Supplementary Table 3. Associations with agreement that GPs hold leadership role in practice sustainability

| **Factor group** | **Variable** | **Class** | **Disagree** | **Agree** | **p** |
| --- | --- | --- | --- | --- | --- |
| Registrar factors | Gender | Male | 101 (52%) | 269 (39%) | 0.002 |
|  |  | Female | 94 (48%) | 415 (61%) |  |
|  | Full or part time employment | Part-time | 44 (23%) | 188 (28%) | 0.15 |
|  |  | Full-time | 150 (77%) | 486 (72%) |  |
|  | Term of training | Term 1 | 92 (47%) | 329 (48%) | 0.79 |
|  |  | Term 2 | 21 (11%) | 83 (12%) |  |
|  |  | Term 3 | 82 (42%) | 272 (40%) |  |
|  | Primary qualification as doctor in Australia | No | 28 (14%) | 165 (24%) | 0.004 |
|  |  | Yes | 167 (86%) | 519 (76%) |  |
|  | Health qualification before medical qualification | No | 167 (87%) | 600 (88%) | 0.56 |
|  |  | Yes | 26 (13%) | 81 (12%) |  |
|  | Non-health qualification before medical qualification | No | 132 (68%) | 458 (67%) | 0.77 |
|  |  | Yes | 61 (32%) | 223 (33%) |  |
|  | Training region | Region 1 | 22 (11%) | 93 (14%) | 0.024 |
|  |  | Region 3 | 6 (3%) | 41 (6%) |  |
|  |  | Region 4 | 36 (18%) | 161 (24%) |  |
|  |  | Region 6 | 79 (41%) | 197 (29%) |  |
|  |  | Region 7 | 52 (27%) | 192 (28%) |  |
|  | Worked at practice previously | No | 167 (87%) | 564 (84%) | 0.40 |
|  |  | Yes | 26 (13%) | 107 (16%) |  |
|  | Response to question regarding Adverse effects of climate change on health | Smaller effect | 101 (78%) | 260 (43%) | <0.001 |
|  |  | Larger effect | 28 (22%) | 339 (57%) |  |
|  | Age | mean (SD) | 33 (6) | 33 (7) | 0.23 |
| Practice factors | Always bulk-bills | No | 117 (60%) | 420 (61%) | 0.71 |
|  |  | Yes | 78 (40%) | 263 (39%) |  |
|  | Practice size | Small | 93 (48%) | 280 (41%) | 0.11 |
|  |  | Large | 101 (52%) | 396 (59%) |  |
|  | Rurality | Major city | 123 (64%) | 395 (58%) | 0.14 |
|  |  | Inner regional | 59 (31%) | 213 (31%) |  |
|  |  | Outer regional/remote/very remote | 11 (6%) | 69 (10%) |  |
|  | Socio-Economic Index for Area (SEIFA) | mean (SD) | 5 (3) | 5 (3) | 0.28 |
